# Complementing the Self-Determination Theory With the Need for Novelty: Motivation and Intention to Be Physically Active in Physical Education Students

**DOI:** 10.3389/fpsyg.2020.01535

**Published:** 2020-07-03

**Authors:** Carlos Fernández-Espínola, Bartolomé J. Almagro, Javier A. Tamayo-Fajardo, Pedro Sáenz-López

**Affiliations:** Department of Integrated Didactics, Faculty of Education, Psychology and Sports Sciences, Universidad de Huelva, Huelva, Spain

**Keywords:** self-determination theory, psychological needs, school education, secondary education, physically active

## Abstract

The theoretical framework of the self-determination theory establishes that the satisfaction of basic psychological needs and more self-determined motivational forms are related to positive behavioral consequences and, therefore, may increase the intention to be physically active in Physical Education students. In this sense, the need for novelty has been proposed as a psychological need by recent scientific evidence, so it is necessary to prove its possible contribution to the prediction of young people’s behavior. Therefore, the main objective of the study was to test a model that analyzed the power of prediction of the satisfaction of the needs for autonomy, competence, relatedness, and novelty as well as the motivation experienced in Physical Education on the intention to be physically active. A questionnaire was administered to 1665 Physical Education students with an average age of 12.43 years (SD = 1.87), of which 835 were boys and 830 were girls. An adaptation of the Spanish educational context of the Basic Psychological Needs in Exercise Scale (BPNES) that includes the need for novelty, the Perceived Locus of Causality Scale (PLOC), and the Intention to be Physically Active Scale (IPAS) was used. Path analysis results showed that the satisfaction of the psychological needs for autonomy, competence, relatedness, and novelty predicted autonomous motivation. On the other hand, the need for autonomy positively predicted controlled motivation, while the satisfaction of relatedness did so negatively. The need for competence and autonomous motivation positively predicted the intention to be physically active in Physical Education students, obtaining an explained variance of 33%. However, controlled motivation was not related in a statistically significant way to the intention to be physically active. In conclusion, the results of the hypothesized model highlight the importance of satisfying all the basic psychological needs (including novelty) and give special emphasis to the need for competence, since it predicts autonomous motivation and the intention to be physically active outside of the educational context.

## Introduction

Despite the multiple benefits that regular physical activity provides in children and adolescents (e.g., Janssen and LeBlanc, 2010), increasing adherence to it and reducing sedentariness among this demographic continues to be a major global health issue (WHO, 2019). In recent years, the study of motivation has been widely applied to the field of physical activity by being considered as that which energizes and gives direction to human behavior (Ryan and Deci, 2017) and, therefore, is very useful as a concept to analyze aspects related to adherence to activity (e.g., Ryan et al., 1997; Teixeira et al., 2012).

As such, one of the principal theories that focuses on the workings of motivational processes in human beings is the self-determination theory (SDT; Deci and Ryan, 1985, 2000; Ryan and Deci, 2017). SDT identifies the existence of three basic psychological needs which are universal and inherent in all individuals. These are autonomy, competence, and relatedness. “Autonomy” refers to a person’s need to see themselves as the origin of their own actions and to experience actions of their own choosing; “competence” refers to the need to control the result and achieve efficiency; and “relatedness” refers to the need to be connected to others (Ryan and Deci, 2017). These three needs explain the regulation of people’s behavior, established on a motivational continuum, which fluctuates between autonomous motivation (i.e., intrinsic regulation, integrated regulation, and identified regulation), passes through more controlled forms of motivation (i.e., introjected regulation and external regulation), and ends with the absence of regulation or amotivation (Deci and Ryan, 2000; Ryan and Deci, 2000, 2017; Vansteenkiste et al., 2010). Intrinsic motivation concerns activities carried out to generate pleasant sensations or for enjoyment and inherent interest. In identified regulation, the person consciously valors an activity and experiences a high degree of volition to act. In integrated regulation, the person valors the activity and finds it to be consistent with other interests. Introjected regulation refers to behaviors performed by avoidance of negative feelings (like shame or anxiety) for failure and by rewards of self-esteem for success. External regulation comprises behaviors which are imposed by a system of rewards and punishments. Finally, amotivation concerns to lacking intentionality (Ryan and Deci, 2020). SDT suggests that the satisfaction of these basic psychological needs is associated with autonomous motivation, while frustration of the same is related to controlled motivation and amotivation (Ryan and Deci, 2017).

As a complement to SDT, Vallerand (1997, 2001) developed the hierarchical model of intrinsic and extrinsic motivation (HMIEM), identifying the existence of three distinct levels of motivation: global (general motivation of the subject), contextual (motivation within a specific context, such as Physical Education classes), and situational (motivation experienced during the course of a Physical Education session or a particular activity). This model established that motivation at any of these levels is influenced by social factors (e.g., the interpersonal style of the teacher); that the perception of the three basic psychological needs mediates the effect between these social factors and motivation; and that motivation brings a series of consequences across a wide variety of contexts (Ryan and Deci, 2017), including in Physical Education (e.g., Van den Berghe et al., 2014). In this way, the scientific literature has shown how satisfaction of the needs for competence, autonomy and relatedness increases the most self-determined types of motivation (Ntoumanis, 2001; Standage et al., 2006; Fin et al., 2019), and this leads to positive behavioral consequences in students, some of which are associated with the practice of physical activity outside of the school context (e.g., the intention to be physically active; Hein et al., 2004; Mouratidis et al., 2008; Lim and Wang, 2009).

Within this context, González-Cutre et al. (2016) recently opened a new line of investigation concerning the functioning of motivational processes. These authors proposed that the need for novelty, understood as the need to experience something that has not been experienced previously or that diverges from the daily routine, could be considered as a new basic psychological need within SDT. In fact, several studies have proposed more new candidate-needs in the last few years (Vansteenkiste et al., 2020).

The fourth mini-theory of SDT, or in other words, the basic psychological needs theory (BPNT), analyze the relations between the basic psychological need satisfactions and frustrations on well-being and the ill-being (Ryan and Deci, 2017). Within BPNT, Ryan and Deci (2017) established six criteria that a candidate need should meet to be including as a basic psychological need: (1) the satisfaction of this candidate must be strongly associated with well-being, health and psychological integrity, while its frustration must be negatively associated with these consequences and positively related with ill-being; (2) it must specify the experiences and contents that lead to well-being; (3) candidate need should be a significant and constant mediator of the relationships between personal and social factors and the motivational and psychological functioning of the individual; (4) any new candidate should act as a growth need and not as a deficit need, meaning that the new basic psychological need must act in accordance with the three existing needs and not as a substitute for one of them when they become frustrated or threatened; (5) it should be a precursor and not a consequence within the motivational process of self-determination theory; and (6) it must work universally for all ages and cultures.

In this sense, Bagheri and Milyavskaya (2020) have proposed and analyzed a similar key-concept to novelty as candidate new. These authors showed how the novelty-variety (personal perception of doing or experimenting something new and the possibility of different combinations) fulfilled some criteria; since novelty-variety was a factor separated from the other needs, the findings did not depend on the age of the participants or novelty-seeking preferences, and when novelty-variety was thwarted, there was a reduction on well-being.

In this line, novelty (excluding the variety-feature) has also fulfilled some criteria. González-Cutre et al. (2016) showed through a confirmatory factor analysis that the need for novelty is a distinct construct to those of autonomy, competence, and relatedness. These results have coincided with recent findings by Trigueros et al. (2019) in a study to validate the Spanish version of the Basic Psychological Needs Satisfaction Scale for Physical Education classes. The confirmatory factor analysis also showed the need for novelty as an independent factor. Likewise, the studies by González-Cutre et al. (2016, 2020) and González-Cutre and Sicilia (2019) have demonstrated how the aforementioned construct is positively associated with the most self-determined types of motivation and with positive consequences, such as life satisfaction, enjoyment, and vitality in Physical Education. González-Cutre et al. (2020) also showed the moderating role of openness to experience in the association between the novelty need satisfaction and well-being.

This existing relationship between the need for novelty and positive consequences has now become the object of study in other contexts. In particular, Birdsell (2018) has recently shown a positive correlation between the satisfaction of the need for novelty and the commitment to and satisfaction of learning English for a group of Japanese students. To this extent, the recent scientific evidence indicates that novelty could be a basic need within BPNT. However, it is still necessary to analyze whether similar benefits emerge at the cognitive, behavioral and physiological level, and in various contexts (Vansteenkiste et al., 2020).

In terms of the role played by the need for novelty with respect to physical activity, little is currently known. In fact, the study by Fernández-Espínola et al. (2020) is the only published investigation on the satisfaction of the need for novelty and its behavioral consequences in relation to the practice of physical activity (the intention to be physically active in the future). The results of this investigation showed that the need for novelty was associated with the intrinsic motivation of Physical Education students and the growth of their intention to be physically active in the future. However, the traditional basic psychological needs were not included in this study. Therefore, further study is required of the role of the need for novelty when combined with the other three psychological needs in a sequence that includes some variable related to the practice of physical activity as a consequence.

**FIGURE 1:**
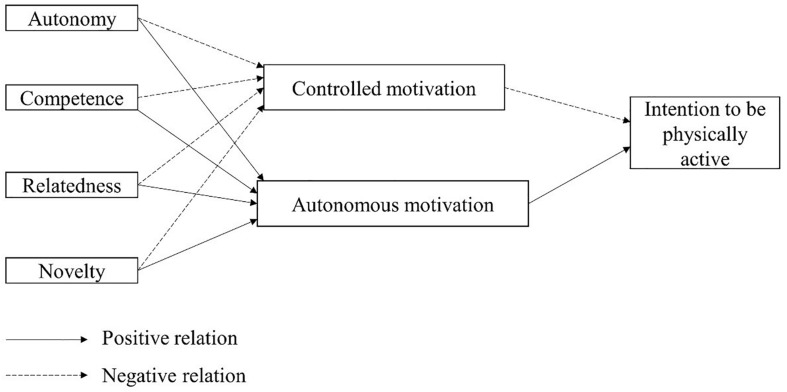
Hypothesized model of the basic psychological needs, with autonomous and controlled motivation and the intention to be physically active.

Thus, taking into account the importance that knowing young people’s intention to be physically active could have for global health, just like the lack of studies that relate the need for novelty with behavioral consequences within SDT, the objective of the present study was to test the interrelations between the need for novelty and the basic psychological needs, autonomous motivation, controlled motivation and the intention to be physically active in the future. Using a model which hypothesizes that the three existing basic psychological needs, as well as novelty, would act as positive predictors of autonomous motivation and as negative predictors of controlled motivation. In turn, autonomous motivation would act as a positive predictor of the intention to be physically active, while controlled motivation would act as a negative predictor of the same (see Figure 1).

## Materials and Methods

### Participants

The sample of this investigation was composed of 1665 students of Physical Education, who were studying between the 5th year of primary school up to the first year of the high school diploma (equivalent to first year of A-level study) and belonged to 15 educational institutions (12 public schools and three private schools) in the province of Huelva (Spain). Specifically, the sample constituted 835 boys and 830 girls, of ages ranging from 10 to 18 years old (*M* = 12.43, SD = 1.87). Convenience sampling was used to select the all the participants. Students received two physical education sessions per week.

### Measures

#### Basic Psychological Needs

The most recent version of the Measurement of Psychological Needs Scale was employed (González-Cutre and Sicilia, 2019), which was adapted from the *Basic Psychological Needs in Exercise Scale* (BPNES; Vlachopoulos and Michailidou, 2006) for the Spanish educational setting, to which the authors added the need for novelty. The scale consisted of a total of 17 items grouped into four aspects (four items per aspect, except for the novelty aspect, which consisted of five items): autonomy (e.g., “I have the opportunity to choose how to exercise”), competence (e.g., “I exercise efficiently”), relatedness to others (e.g., “I feel very comfortable when I exercise with friends”) and novelty (e.g., “I think that I often discover new things”). The preceding phrase was “In my Physical Education classes…”. The responses were scored using a Likert scale with five options, from 1 (corresponding to *Totally agree)* to 5 (corresponding to *Totally disagree)*. The internal consistency of this scale was 0.83 for the need for novelty, 0.74 for the need for relatedness, 0.73 for the need for autonomy, and 0.70 for the need for competence. Factorial validity of the Psychological Needs Scale was examined using confirmatory factor analysis (CFA), which offered acceptable fit indices (χ^2^ = 417.21, *p* = 0.00, χ^2^/g.l. = 3.69, CFI = 0.92, IFI = 0.92, TLI = 0.91, SRMR = 0.05, RMSEA = 0.06).

#### Autonomous and Controlled Motivation

The factors of intrinsic regulation, identified regulation, introjected regulation and external regulation were used from the Perceived Locus of Causality Scale in Physical Education, tested in the Spanish context by Moreno-Murcia et al. (2009) from the *Perceived Locus of Causality Scale* (PLOC; Goudas et al., 1994). Each factor is composed of 4 items: intrinsic regulation (e.g., “Because physical education is fun”), identified regulation (e.g., “Because I want to learn sporting skills”), introjected regulation (“Because I would feel bad about myself if I didn’t do it”) and external regulation (e.g., “Because I’ll have problems if I don’t do it”). The responses were scored using a Likert scale, with a scoring system range from 1 (*Totally disagree)* to 7 (*Totally agree)*. The phrase preceding the items was “I participate in Physical Education classes…”. The internal consistency was 0.78 for intrinsic regulation, 0.78 for identified regulation, 0.69 for introjected regulation, and 0.70 for external regulation. The data from this study revealed adequate psychometric properties of the PLOC in PE using CFA (χ^2^ = 520.60, *p* = 0.00, χ^2^ / g.l. = 3.25, CFI = 0.92, IFI = 0.92, TLI = 0.91, SRMR = 0.06, RMSEA = 0.06). In order to calculate autonomous motivation, the sum of intrinsic regulation subscale weighted by two and of identified regulation subscale was used. Similarly, in order to calculate controlled motivation, the sum of external regulation scale multiplied weighted by two and of introjected regulation scale was used (Hagger et al., 2014).

#### Intentionality to Be Physically Active

The adapted and translated (into Spanish) version (Moreno-Murcia et al., 2007) of *Intention to be Physically Active Scale* (IPAS) by Hein et al. (2004). The scale was introduced with the phrase “With respect to your intention to practice some form of physical activity…”. The scale was composed of five items in order to measure the intention to be physically active (e.g., “I regularly practice sport in my free time”). The responses were scored using a Likert scale, ranging from 1 (*Totally disagree)* to 5 (*Totally agree*). Cronbach’s alpha was 0.77. The CFA showed acceptable fit indices (χ^2^ = 24.45, *p* = 0.00, χ^2^ / g.l. = 4.89, CFI = 0.99, IFI = 0.99, TLI = 0.98, SRMR = 0.02, RMSEA = 0.08).

### Procedure

The present study was developed in accordance with current legislation in Spain concerning investigative studies of people and in line with the ethical principles of the American Psychological Association (2020). Firstly, the permission of the Ethics Committee of Biomedical Investigation of Andalusia (Spain) was obtained. The next measure completed to enable data collection was to establish contact with the management team of each of the educational institutions, with the intention of informing them about the aims of our investigation and to request their cooperation. Subsequently, given that the sample consisted of subjects who were minors, authorization in the form of a written signature was requested from their legal guardians so that they could participate in the study. Once authorization was granted, the questionnaires were distributed in the presence of the principal researcher, who gave a brief explanation of the objectives of the study, set out a series of guidelines on how to complete the questionnaires and addressed any issues that arose from the reading and comprehension of some of the items, emphasizing anonymity and the importance of providing honest answers. In general, the time required to complete the questionnaire was 15 minutes.

### Data Analysis

Firstly, the data matrix was filtered, followed by an analysis of the reliability of the data and calculation of the variables. Then, descriptive statistics of said variables and bivariate correlations were calculated. Moreover, a path analysis was completed with the aim of analyzing the hypothesized predictive relationships between the study variables using the method of estimation of maximum authenticity. Regarding indirect effects, Preacher and Hayes’ (2008) methods of multiple mediation were used, generating the limits of confidence for the indirect effects through bootstrapping. In order to verify the validity of the model, the following goodness-of-fit indices were taken into account: the CFI (*Comparative Fit Index*), the IFI (*Incremental Fit Index*), the TLI (*Tucker Lewis Index*), the ratio between chi-squared and degrees of freedom (χ^2^ /g.l.), the SRMR (*Standardized Root Mean Square Residual*), and RMSEA (*Root Mean Square Error of Approximation*) combined with its confidence interval (CI) of 90%. The aforementioned goodness-of-fit indices are considered acceptable when the values of CFI, IFI, and TLI are greater than 0.90 (Hu and Bentler, 1995), 0.08 or lower for RMSEA (Browne and Cudeck, 1993) and SRMR (Hu and Bentler, 1999), and between 2 and 3 for χ^2^ /g.l. (Schermelleh-Engel et al., 2003). This analysis was completed with the statistical programs SPSS 23.0 and Amos 23.0.

## Results

### Descriptive Analysis and Bivariate Correlations

In Table 1, the descriptive statistics (mean, standard deviation, skewness and kurtosis) and bivariate correlations are shown. In terms of the mean scores of the motivational regulations, identified regulation, followed by intrinsic motivation, showed the highest value. Autonomous motivation showed higher mean score than controlled motivation. With respect to the mean scores of the basic psychological needs, the need for relatedness, followed by the need for competence, displayed the highest score.

**TABLE 1:** Descriptive statistics and bivariate correlations of the study variables.

**Variables**	**RG**	***M***	**SD**	**S**	**C**	**1**	**2**	**3**	**4**	**5**	**6**	**7**	**8**	**9**	**10**	**11**
1. Autonomy	1–5	3.40	0.86	–0.27	–0.30	–	0.53**	0.34**	0.64**	0.47**	0.47**	0.33**	0.08**	0.50**	0.18**	0.33**
2. Competence	1–5	3.96	0.76	–0.67	0.01	–	–	0.46**	0.54**	0.59**	0.60**	0.27**	–0.03	0.63**	0.08**	0.52**
3. Relatedness	1–5	4.18	0.79	–0.91	0.16	–	–	–	0.46**	0.43**	0.41**	0.11**	−0.07**	0.45**	–0.17	0.34**
4. Novelty	1–5	3.83	0.89	–0.76	0.19	–	–	–	–	0.54**	0.55**	0.27**	0.01	0.58**	0.11**	0.37**
5. Intrinsic R	1–7	5.83	1.15	–1.21	1.36	–	–	–	–	–	0.76**	0.26**	−12**	0.98**	0.00	0.48**
6. Identified R	1–7	5.84	1.13	–1.33	1.86	–	–	–	–	–	–	0.36**	–0.02	0.89**	0.11**	0.53**
7. Introjected R	1–7	4.39	1.43	–0.21	–0.54	–	–	–	–	–	–	–	0.51**	0.31**	0.75**	0.24*
8. External R	1–7	3.87	1.54	–0.06	–0.79	–	–	–	–	–	–	–	–	−0.09**	0.95**	–0.04
9. Autonomous M	3–21	17.50	3.26	–1.30	1.73	–	–	–	–	–	–	–	–	–	0.04	0.53**
10. Controlled M	3–21	12.12	4.00	–0.04	0.74	–	–	–	–	–	–	–	–	–	–	0.05*
11. Intention	1–5	4.22	0.80	–1.18	0.96	–	–	–	–	–	–	–	–	–	–	–

* p < 0.05 ** p < 0.01; M, mean; SD, standard deviation; S, skewness; C, kurtosis; R, regulation; RG, range; M, motivation.

In turn, in the correlation analysis it was observed that all the basic psychological needs correlated positively and significantly with the intention to be physically active and with all types of motivation, except for external regulation, where the need for autonomy correlated positively and significantly, whilst the need for relatedness correlated negatively and significantly. Regarding the correlation of motivational regulations and the intention to be physically active, all types of motivation correlated positively and significantly, except for external regulation.

### Path Analysis

The results of the hypothesized model, where autonomous motivation and controlled motivation, respectively interceded positively and negatively between the satisfaction of the basic psychological needs and the intention to be physically active, showed that the need for novelty and the three basic psychological needs were positive and significant predictors of autonomous motivation, and that the latter also behaved as a positive predictor of intention. In turn, controlled motivation was only predicted significantly by the needs for relatedness (negatively) and autonomy (positively), and contrary to what was expected, behaved as a positive but insignificant predictor of intention. However, this model did not show adequate goodness-of-fit indices, χ^2^ = 144.99, *p* < 0.05, χ^2^ /g.l. = 28.99, CFI = 0.97, IFI = 0.97, TLI = 0.85, SRMR = 0.043, RMSEA = 0.13, 90% CI [0.11, 0.14].

In this regard, with the aim of obtaining an improved fit of the model, the three relationships that showed an insignificant weight of regression (between the needs for competence and novelty with controlled motivation, and between the latter and the intention to be physically active) were eliminated, and the modified indices of the model were then observed. These suggested that the model could be improved, adding a direct relationship between the need for competence and the intention to be physically active. Thus, considerable improvements were achieved for the goodness-of-fit indices: χ^2^ = 14.59, *p* < 0.05, χ^2^/g.l. = 2.09, CFI = 0.99, IFI = 0.99, TLI = 0.99, SRMR = 0.011, RMSEA = 0.026, 90% CI [0.005, 0.044].

**FIGURE 2:**
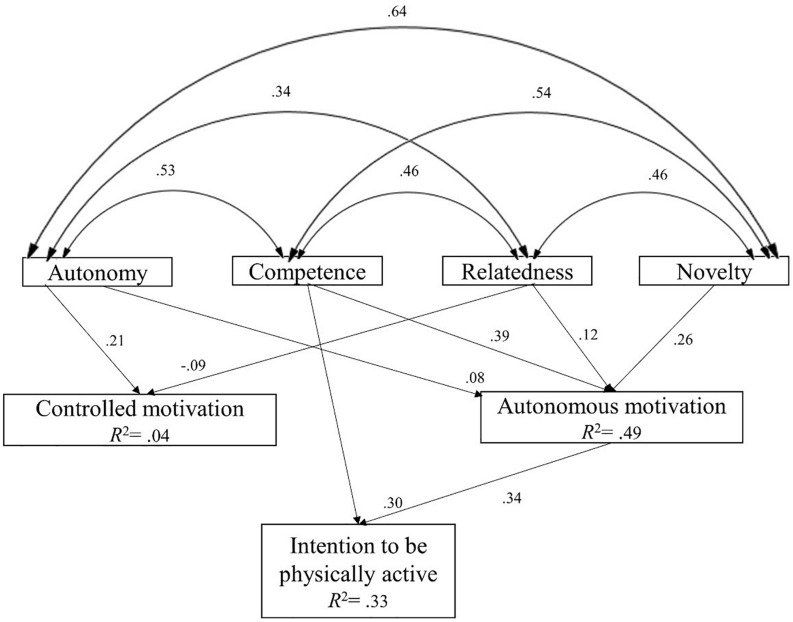
Final path model of existing relationships between the four basic psychological needs, autonomous motivation, controlled motivation, and the intention to be physically active in the future. *R*^2^ = explained variances.

The results of this model (see Figure 2) showed that satisfaction of the needs for autonomy (β = 0.08, *p* < 0.001), competence (β = 0.39, *p* < 0.001), relatedness to others (β = 0.12, *p* = 0.001) and novelty (β = 0.26, *p* < 0.001) were positive and significant predictors of autonomous motivation. In turn, with regard to controlled motivation, the need for autonomy acted as a positive predictor (β = 0.21, *p* < 0.001), and the need for relatedness as a negative predictor (β = −0.09, *p* < 0.001).

With respect to the intention to be physically active, this was predicted by autonomous motivation (β = 0.34, *p* < 0.001) and the need for competence (direct effect: β = 0.30, *p* < 0.001; indirect effect: β = 0.13, *p* < 0.001). Likewise, other indirect effects of intention to be physically active were found with respect to the need for autonomy (β = 0.03, *p* < 0.001), novelty (β = 0.09, *p* < 0.001) and relatedness to others (β = 0.04, *p* < 0.001). The explained variances were 49% for autonomous motivation, 4% for controlled motivation, and 33% for the intention to be physically active.

## Discussion

The objective of this study was to test the interrelationships between the need for novelty and the basic psychological needs, as well as motivation and the intention to be physically active in the future, through the use of a model which hypothesized that all the needs would act as positive predictors of autonomous motivation, and as negative predictors of controlled motivation. Equally, autonomous motivation would act as a positive predictor of the intention to be physically active, while controlled motivation would act as a negative predictor of the same. The results obtained have demonstrated partial support for the hypothesized model.

Firstly, the need for novelty and the three existing basic psychological needs predicted autonomous motivation both positively and significantly. These results were consistent with our model and the findings of González-Cutre and Sicilia (2019), Trigueros et al. (2019), and González-Cutre et al. (2020), where the types of autonomous motivation were positively predicted by the satisfaction of the four needs. This supports the idea that the need for novelty acts in synergy with the other three basic psychological needs, thus fulfilling one of the criteria established by Ryan and Deci (2017). For these authors, the fourth criterion that a proposed need must fulfill in order to be included in BPNT is that it acts as a growth need and not as a deficit need, meaning that the new basic psychological need must act in accordance with the three existing needs and not as a substitute for one of them when they become frustrated or threatened.

On the other hand, with respect to controlled motivation, the results obtained were not the same as those hypothesized. Only the needs for autonomy and relatedness were associated with it. The need for relatedness predicted controlled motivation negatively, while, contrary to what was expected, autonomy behaved as a positive predictor of the same. Currently, it is possible to compare these results only with the findings of González-Cutre et al. (2020), because this is the only study to have included controlled motivation within a similar motivational sequence. The results of the investigation by González-Cutre et al. (2020) showed that the need for competence acted as a negative predictor of controlled motivation, in accordance with the hypothesis of the proposed model. The satisfaction of a basic psychological need as a predictor of controlling motivation may be due to the fact that supporting autonomy, competence, and relatedness is positively correlated with introjected regulation and negatively and weakly correlated with external regulation (Vasconcellos et al., 2019).

Finally, with regard to the intention to be physically active, the results partially supported those proposed in the hypothesized model. In this regard, intention was predicted positively and directly by autonomous motivation, while no significant relationship was found with controlled motivation. These results agree with previous investigations (Hein et al., 2004; Taylor et al., 2010), where the most self-determined types of motivation behaved as positive predictors of the intention to be physically active in the future, providing evidence which supports the idea that what happens in Physical Education classes can affect students’ behavior in their free time (Hagger et al., 2003; Hagger and Chatzisarantis, 2012; Ferriz et al., 2016). Moreover, it was found that satisfaction of the need for competence was the only one that directly affected this variable. In the study by González-Cutre et al. (2020), the need for competence was also the only one to present direct effects on the two consequences concerned (enjoyment and vitality). In this line, the recent systematic review and meta-analysis by Vasconcellos et al. (2019) has showed that the need for competence presents higher direct effects on adaptive outcomes than the need for autonomy or need for relatedness. As a result, it seems that the need to control and succeed in tasks by themselves could be a key factor in determining whether the practice of physical activity during childhood and adolescence is continued into adult life.

Likewise, the intention to be physically active was also indirectly predicted by the needs for autonomy, relatedness and novelty, the last of which presented the largest indirect effect. These results support the mediating role played by motivation in the existing relationship between the satisfaction of basic psychological needs, including the need for novelty, and its consequences, thus partially fulfilling another of the criteria established by Ryan and Deci (2017) to be able to include a new need in their theory. For these authors, any new need should behave as a constant and significant mediator of relationships between personal and social factors and the motivational functioning and psychology of the individual.

On the other hand, the principal limitations found in the study must be addressed, as well as future lines of investigation. Firstly, being of cross-sectional design, this study has not been able to analyze whether the satisfaction of the need for novelty is associated with positive consequences over a long period of time. It would be interesting for future studies to utilize a longitudinal design in order to explore this. Secondly, no social factors (e.g., interpersonal style of the teacher) have been measured in this study, and so it has not been able to assess the effect of measuring satisfaction of the need for novelty against social factors, motivation and their consequences. Future studies should analyze the role played by the need for novelty together with the three basic psychological needs in the complete sequence proposed by the theory of self-determination. Thirdly, neither integrated regulation nor amotivation was measured in this study. Finally only the relations of the satisfaction of the basic psychological needs have been analyzed in this study. It would be interesting for future investigations to explore the importance that the frustration of these needs has upon the intention to be physically active in the future.

## Conclusion

In conclusion, the results of the hypothesized model have shown the importance of satisfying the need for novelty together with the other basic psychological needs (with special emphasis on the need for competence) in Physical Education classes, because this predicts the autonomous motivation and intention to be physically active of students in the future, outside of the school context.

As the results of the present study indicate, if teachers of Physical Education wish to increase the intention of their students to be physically active in the future, they must consciously try to generate a learning atmosphere in their classes that satisfies both the traditional basic psychological needs and the need for novelty (setting novel tasks and challenges that go beyond the daily routine), because said satisfaction predicts autonomous motivation and, consequently, grows the intention to practice physical activity in the future, outside of the educational context. In line with what is proposed by González-Cutre and Sicilia (2019), we suggest that, in order to introduce novel themes in Physical Education classes, teachers could apply methodologies unrelated to traditional approaches (e.g., gamification), use different materials, introduce the use of TIC, take students away from the school in order to practice physical activity (e.g., climbing and hiking), or call in experts so that students can take part in activities that are different to those typically taught in the educational center (e.g., parkour and zumba). Furthermore, the results of the study highlight the satisfaction of the need for competence above the other needs, because this directly predicts intention. Therefore, it is necessary for teachers to put special emphasis on proposing motor tasks and challenges that are achievable for the students, in order to help them to establish personal improvement as a principal objective, to allow them sufficient time to practice, to offer fundamentally positive encouragement, etc.

## Data Availability Statement

The raw data supporting the conclusions of this article will be made available by the authors, without undue reservation.

## Ethics Statement

The studies involving human participants were reviewed and approved by the Portal de Ética de la Investigación Biomédica de Andalucía (Spain) (https://www.juntadeandalucia.es/salud/portaldeetica/). Written informed consent to participate in this study was provided by the participants’ legal guardian/next of kin.

## Author Contributions

CF-E was responsible for conducting the study process, collaborating in the design of the study, collecting the data, conducting a preliminary analysis, and writing part of the manuscript. BA collaborated in the design of the study, was responsible for carrying out the analysis of the data and writing the results, collaborated in writing various sections of the manuscript, and has reviewed the final version. JT-F collaborated in the writing of the article and reviewed the final version. PS-L had primary responsibility for the design of the study, collaborated in the writing of the article, and reviewed the final version. All the authors collaborated significantly in the preparation of the manuscript and have given their approval to the final version of the article.

## Conflict of Interest

The authors declare that the research was conducted in the absence of any commercial or financial relationships that could be construed as a potential conflict of interest.
